# Perceived acceptability of a prototype toolkit to support patients and informal caregivers to express their perspectives in palliative care conversations^☆^

**DOI:** 10.1016/j.pecinn.2025.100387

**Published:** 2025-03-17

**Authors:** Annet Olde Wolsink-van Harlingen, Jan Jukema, Kris Vissers, Madeleen Uitdehaag, Jeroen Hasselaar, Leontine Groen-van de Ven

**Affiliations:** a Saxion University of Applied Sciences, Research Group Smart Health, Deventer/Enschede, Netherlands; b Radboudumc University Medical Centre, Department of Anaesthesiology, Pain and Palliative Medicine Nijmegen, Netherlands; c Dimence Mental Health Group, Deventer, Netherlands; d Saxion University of Applied Sciences, Saxion Research and Graduate School, Deventer/Enschede, Netherlands; e Radboud University Medical Centre, Department of Primary Care, Nijmegen, Netherlands; f Nivel Netherlands Institute for Health Services Research, Utrecht, Netherlands; g Windesheim University of Applied Sciences, Research Group Living Well With Dementia, Zwolle, Netherlands

**Keywords:** Chronic obstructive pulmonary disease, Chronic heart failure, Palliative care, Patient perspective, Design thinking, Acceptability, Toolkit

## Abstract

**Background:**

Patients and informal caregivers experience challenges to express their personal perspectives in conversations with healthcare professionals (HCPs). A prototype toolkit, which consists of a hardcopy version and a website, was developed to address their challenges. The aim of this study is to gain insight into the perceived acceptability of this prototype toolkit.

**Method:**

Patients and informal caregivers end users and HCPs participated in semi-structured individual or group interviews. This resulted in two databases of qualitative data which were thematically analysed.

**Results:**

Twenty-two end users and twelve HCPs participated in this study. There is appreciation for the content and use of the prototype toolkit, with the hardcopy version of the toolkit being valued more than the website. Moreover, the use of the toolkit may strengthen end users' power and control and may support HCPs in tailoring communication and care. End users and HCPs recommendations for implementation are to further develop the prototype toolkit, provide HCPs with information, instruction and support and create facilitating conditions in healthcare.

**Conclusion:**

High appreciation of the hardcopy version and the practical value are positive indicators of end users'and HCPS perceived acceptability of the prototype toolkit. However, the content of the toolkit is experienced as being too extensive, with the hardcopy version experienced as being incomplete without the website and the website is experienced as being too complicated to use. Further development and testing of the prototype toolkit is required to increase its acceptability by end users and HCPs.

**Innovation:**

In this study a Design Thinking approach was used to test study the acceptability of a prototype toolkit by endusers and HCPs. This approach can contribute to a succesfull implementation of the toolkit and its effectiveness.

## Highlights

• High appreciation of hardcopy version prototype toolkit.
• High appreciation of practical value prototype toolkit.
• The use of the toolkit may strenghten power and control of endusers.
• The use of the toolkit may support healthcare professionals to tailor communication and care.

## 1 Introduction

Patients with chronic obstructive pulmonary disease (COPD) and chronic heart failure (CHF) and their informal caregivers (ICs) can benefit from a person-centred palliative care approach to improve both their quality of life and care [[1], [2], [3]]. Person-centred palliative care can be considered as a shared responsibility of patients, ICs and healthcare professionals (HCPs) based upon the knowledge and expertise of those involved. Patients and ICs can become experts in living with the disease whereas HCPs are expected to be experts on disease, treatment and care [[4], [5], [6]]. Knowing the personal perspectives of patients and ICs, what they value, need and prefer in their life, health and care, is an important pre-requisite to enable HCPs to provide person-centred palliative care [[7], [8], [9]].

However, not every patient and IC is willing or able to express their personal perspective in conversations with HCPs [[10], [11], [12], [13], [14]]. Due to many different personal, interactional and organisational factor patients, ICs and HCPs consider it challenging to personalise their conversations during consultations [[10], [11], [12], [13], [14], [15], [16]]. Patients and ICs experience these challenges even more so when there is a lack of empathy and a good connection with their HCP. Moreover, HCPs experience that conversation can be difficult for them when patients are passive, closed, and unable to understand or recall the information or when they have unrealistic expectations [16]. This inability and behaviour of patients is associated with lower levels of health literacy and social skills [11].

Many tools exist for patients and ICs to help them prepare for conversations with their HCPs and to facilitate these conversations [[17], [18], [19], [20], [21]]. However, these tools are often not used because for example, patients, ICs and HCPs are unaware that they exist, it is hard for patients and ICs to assess their relevance and quality (e.g. up-to-date evidence-based information from transparent and unbiased sources which can be understood by lay people [22]) and/or it is challenging to integrate these tools in the care process, for example because HCPs experience time constraints [18,20,22,23]. Since it is important to provide patients with COPD or CHF and their ICs, (hereafter end users), with relevant, high quality and accessible tools supporting them in exploring and expressing their perspective in conversations with HCPs, a prototype toolkit (MijnBlik) was developed in co-creation with end users and HCPs [15]. This prototype toolkit consists of a hardcopy version (tin box) and a corresponding website bringing existing (Dutch) tools from different sources together in one place [15]. Tools were selected based on a set of defined quality criteria; relevance in relation to experienced challenges, reliability by means of being provided by trustworthy organisations, specific for target population and setting, readable and understandable by lay people [15].

Succesful evaluation and implementation of the prototype toolkit depends, among other factors, on its acceptance by its intended receivers (end users) and intended deliverers (HCPs) [24,25]. Since we can view this prototype toolkit as a technology, the conceptualisation of acceptability according to the Technology Acceptance Model (TAM) has been initiated [[26], [27], [28]]. Acceptability of a new technology is understood as a three stage proces; 1) determinants like system characteristics, facilitating conditions, individual differences and social influences lead to the development of two beliefs of the new technology: a) it's perceived usefulness (PU) and b) it's perceived ease-of-use (PEOU). These two beliefs influence the 2) intention to use and the 3) actual usage behaviour [27,28].

Insight into the perceived acceptability of the prototype toolkit by end users and HCPs is necessary to decide whether the prototype toolkit needs to be adjusted prior to large scale evaluation and implementation [29]. Therefore, we want to explore how end users and HCPs perceive the acceptability of the prototype toolkit in view of the following questions: 1) What is and is not, appreciated about the prototype toolkit? 2) What is the perceived practical value of the prototype toolkit? 3) What are recommendations for the implementation of the prototype toolkit in conversations and organisational settings?

## 2 Method

### 2.1 Design

This study was part of a broader research and innovation project named EMPATIE which stands for “EMpowerment of PATIEnts and informal caregivers” performed in the eastern part of the Netherlands [15]. The aim of this project was to develop a supportive prototype toolkit for end users enabling them to express their personal perspective in conversations with HCPs. The first four phases of Design Thinking (DT) empathize, define, ideate, prototype, [30,31] were used to develop a prototype toolkit in co-creation with end users and HCPs [15]. The current study reports the findings concerning the fifth phase of DT wherein the acceptability of the prototype toolkit was tested by end users and HCP's prior to the evaluation and implementation of the prototype toolkit in clinical practice. From April to November 2021 we collected in-depth qualitative data via semi-structured individual and group interviews with end users and HCP's [32].

### 2.2 Prototype toolkit

The prototype toolkit consists of a hardcopy version and a corresponding website. The hardcopy version was developed to introduce the tools whereas the website was developed to provide easy access to free, available online tools (mijn-blik.nl). This means that the hardcopy version contains a description of the tools and that the website is required to gain access to the freely available online tools. The tools were selected based upon the selection criteria that were revealed in the first study of the project [15]. The hardcopy version is a tin box which contains an information and instruction sheet and 42 cards. Each card gives a description of a tool or some general information about the tool. The cards are bound together in four fans representing different topics based upon the user's needs and wishes [15]; namely 1) I want to say what is on my mind, 2) I want to know which questions I can ask, 3) I want to participate in the conversation about my treatment and care, 4) I want to talk about the future. The first page of the website is a welcome page with a short introduction and four tiles each of which refers to one of the four topics respectively. The colours and the pictures of the tiles correspond with the paper fans and cards of the hardcopy toolkit. End users can use the prototype toolkit to prepare themselves independently for conversations with HCPs in a self-paced manner at home.

### 2.3 Participants

#### 2.3.1 End users

End users needed to be able to understand and speak Dutch without having any mental or speaking disorders such as dementia or aphasia. They also needed to be under periodical supervision of a medical specialist, general practitioner, nurse or nurse practitioner.

Different approaches were used to recruit future end users as participant for this study. Firstly, HCPs participating in the previous phases of the EMPATIE-project, recruited end users during scheduled consultations. Secondly, two national patient organisations for COPD and CHF patients and one informal caregiver organisation were asked to send out a recruitment announcement to their members. Thirdly, physical therapists specialized in rehabilitation of patients with COPD or CHF contributed to the recruitment by personally asking patients and ICs whether they were willing to participate. Fourthly, an article about the prototype toolkit was published in a local newspaper to invite people interested in receiving and testing the toolkit to contact us by mail or phone.

When end users were interested in participating in the study, researcher AO provided additional information about the project, the prototype toolkit and an individual or group interview. After verbal informed consent, AO collected demographic information and made an appointment for an individual or group interview. AO also sent a package with an information letter, an informed consent form, a return envelope and the toolkit to the home addresses of participants at least two weeks in advance of the scheduled interview. During these two weeks participants could use the toolkit in a self-paced manner at home correspondings with its intended use prior to use during conversations with HCPs. Selection for individual or group interview was based upon individual preference. The aim was to recruit at least twenty end users with an equal distribution of 1) patients and ICs and 2) diagnosis COPD and CHF.

#### 2.3.2 Healthcare professionals

HCPs working with patients with COPD or CHF were also eligible for this study. Medical specialists and nurse practitioners working in a hospital setting, general practitioners (GPs) and nurses working in a general practice and nurse practitioners providing care at home could all be included in the study.

HCPs were recruited using different strategies: 1) HCPs, participating in (previous studies of) the EMPATIE-project, recruited colleagues within their network, and 2) a coordinator of the palliative care network approached HCPs in person and via a digital announcement in that network.

HCPs interested in participating were contacted by researcher LG or AO by phone and the same procedure for inclusion was used as described for end users. They also received an information package containing the hardcopy toolkit at least one week before the individual or group interview. During this week HCPs could assess the prototype toolkit on its intended use during conversations with end users. HCPs all provided verbal informed consent before the start of the interview.

We aimed to recruit at least fifteen HCPs with a balanced mixture of HCPs working in a general practice, hospital or providing care at home. We strived for an equal distribution of HCPs working with patients with COPD or CHF.

### 2.4 Data collection

The research questions in this study focus on system characteristics of the toolkit and facilitating conditions (determinants) in relation to the beliefs PU and PEOU (see Table 1).

**Table 1:** Relation between research questions, determinants and beliefs.

Research questions	Determinants	Beliefs
1. What is and is not, appreciated about the prototype toolkit?	System characteristics	PU + PEOU
2. What is the perceived practical value of the prototype toolkit?		PU
3. What are the recommendations for the implementation of the prototype toolkit in conversations and organisational settings?	Facilitating conditions	PU + PEOU

Semi-structured individual and group interviews were performed from April 2021 until November 2021 by researchers LG or AO. A topic list for end users and one for HCPs was used for individual interviews (see Appendix A). For group interviews a script was used with questions and statements derived from these lists. The semi-interviews were performed by phone or video-calling due to Covid-19 restrictions during the inclusion period and face-to-face as soon as these restrictions were lifted. End users were interviewed at home and HCPs at home or at their office.

### 2.5 Data-analysis

All individual and group interviews were audio-recorded and transcribed verbatim. The transcripts were thematically analysed by researcher AO using ATLAS.ti 22. The interviews with end users and HCPs were approached as two datasets. These were analysed separately using line by line coding. Initial codes were categorised and generated into themes by AO. Multiple individual discussion between AO and researchers MU, JJ and LG were used to further review and develop these initial codes, categories and themes. Further refinement was realized through multiple discussions between all participating researchers.

### 2.6 Ethics

The Ethical Committee Twente declared that the Clinical Research Involving Human Subjects Act was not applicable to this research and innovation project. This study was executed according to the Declaration of Helsinki [33]. Informed consent procedures of participants were performed according to good clinical practice guidelines. Saxion University of Applied Sciences' cloud was used to store data conform the description in a data-management plan which was approved by the subsidy provider. This storage protects against loss, damage, unauthorized access and the digital aging of the data files.

## 3 Results

### 3.1 Participants

A total of 22 end users (17 patients, 5 ICs) and 12 HCPs participated in this study. For demographic information of the participants see Table 2. The five participating ICs were not related to any of the patients participating in this study, but were the partner (*n* = 2), an adult child (n = 2), and an adult sister (*n* = 1) of a patient.

### 3.2 Data-collection

Twenty six individual interviews and three group interviews were performed (Table 2). Three interviews with patients were performed in the presence of a spouse not participating in this study. One HCP participated in both an individual face-to-face interview and an online group interview. The median duration of the individual and group interviews with end users was 49 min (range 33–70) and with HCPs 37 min (range 10–64).

**Table 2:** Demographic and method information participants.

Characteristic	Specification	End users (*n* = 22)	Characteristic	Specification	HCPs^1^ (*n* = 12)
**Demographic**					
Diagnosis Patient	COPD	12	Profession	General Practitioner	2
	CHF^2^	5		Nurse	1
	COPD and CHF	5		Nurse Practitioner	
				- COPD	3
				- CHF	4
				- Older adults	2
Gender	Female	15	Gender	Female	11
	Male	7		Male	1
Age	<60	2	Age	<40	4
	60–69	10		40–49	3
	70–79	7		50–59	2
	80–89	3		>60	1
	Missing	0		Missing	2
Level of education^3^	Secondary education	7	Setting	General Practice	4
	Preparatory vocational education (low and medium level)	11		Hospital	3
	Higher Professional education or University	4		Home Care	5
Years since diagnosis patient	<1	5	Years of experience in profession	<6	0
	3–5	4		6–10	3
	6–10	3		>10	4
	>10	10		Missing	5
**Method**					
Type of Interview	Individual	18	Type of interview	Individual	8⁎
	Group	4		Group	5⁎
Form of Interview	Face to face	5	Form of interview	Face to face	1⁎
	Phone call	9		Phone call	3
	Video call	8		Video call	9⁎

1 HCPs = healthcare professionals.

2 CHF = chronic heart failure.

⁎ one HCP participated first in an individual interview ((face to face) and later in a group interview (video-call).

### 3.3 Results

#### 3.3.1 Appreciation of the prototype toolkit

What end users and HCPs like and dislike about the prototype toolkit has been related to three themes: 1) form, 2) content and 3) use. These three themes contain respectively six, five and six categories.

What is appreciated about the form is the original and handy design of the hardcopy version, its inviting appearance and accessible lay-out. However, participants find the tin box too big since it doesn't fit through a letterbox or in a handbag or workbag. They experience the hardcopy version as being incomplete without the corresponding website since it only contains references to websites. They also perceive that the website reduces the usability of the prototype toolkit because not every end user is sufficiently skilled, or finds it too much trouble to use a computer or tablet, especially older adults and severely ill patients (see Appendix B).

What is liked about the contents are the four topics of the fans with cards that get to the heart of the matters of care and the inclusion of appealing tools on the website. In spite of this, end users and HCPs experience the content of the prototype toolkit as too comprehensive. Some end users expressed that the content of the prototype toolkit does not meet their needs because they prefer concrete information, do not feel the need to visit the website or have already discussed everything with their HCP. HCPs on the other hand believe that the sequence of topics presented does not correspond with the course of the diseases. They wonder when they should introduce the toolkit and whether they need to introduce and deliver the toolkit in one go or step by step (see Appendix B).

What is appreciated about the use is that the prototype toolkit is in general easy to use, can be used in a self-paced manner and has a broad application namely it can be used by both end users and HCPs. End users also perceive that the prototype toolkit can be used during the whole course of the diseases. However, end users and HCPs express that specific aspects of the prototype toolkit are too complex and they perceive that it takes too much time and effort to use. These aspects are primarily related to the use of the website and the introduction of the prototype toolkit and its use during consultations (see Appendix B).

#### 3.3.2 Practical value of the prototype toolkit

This study resulted in two themes related to the assigned practical value. The use of the prototype toolkit may 1) strengthen end users' power and control, and 2) support HCPs to align with end users.

The first theme ‘strengthen end users’ power and control’ contains seven categories. First, the use of the prototype toolkit can provide end users with insight into the disease and its process, conversations with HCPs and possibilities of future care. Second, it can create end users' awareness of what to think about, what questions to ask and to whom, and what to discuss in consultations with HCPs. End users also describe that it creates awareness on how to communicate with HCPs, whereas HCPs mention aspects related to what end users can do by themselves and what is preoccupying them. Third, the use of the prototype toolkit can be experienced as confrontational, and especially the theme “talking about the future”, and can lead to certain thoughts and feelings. Fourth, it makes end users think about conversations with their HCPs and their future including end of life. Fifth, especially according to HCPs, it may provide incentives to act, such as talking to others and preparing for consultations with HCPs. Sixth, the use of the prototype toolkit can support dialogue between patients and relatives which may lead to a better understanding of each other. Finally, according to HCPs, the use of the prototype toolkit may support conversations between end users and HCPs and decision making because it may lead to asking the right questions, discussing certain topics and taking the right decisions (see Appendix B).

The second theme for using the prototype toolkit is that it may support HCPs in aligning with end users. This theme contains three categories. First, it may support dialogue between end users and HCPs because among other things, it may contribute to shared responsibility for the content of the conversation and maylead to more active participation of end users in the conversation. Second, it may support HCPs to give more power to the end users and emphasize self-management. Third, it may help HCPs to tailor care because they may respond to the care question of end users and personalise their care (see Appendix B).

#### 3.3.3 Recommendations for adjustments and implementation of the prototype toolkit

End users and HCPs give four recommendations in relation to the adjustments and implementation of the prototype toolkit in the organisational setting.

First, they recommend adjusting the content to make it suitable for a broader target population than COPD or CHF, make it simpler and more convenient to use, make the tin box usable without the need to visit the corresponding website. They also recommend making the tin box smaller and laminating the cards.

Secondly, HCPs express that they need additional information, instruction and support in advance in order to be able to introduce and use the toolkit. They would like to receive more information about why the toolkit has been developed, which HCP is going to deliver it and to whom, when the toolkit can be introduced and delivered, what is handed over and how to integrate toolkit use into conversations with end users. They also expressed the need for preparation and support enabling them to start using the toolkit. Both end users and HCPs state that a trustful relationship is a prerequisite to introducing the toolkit and HCPs express that it is important to clarify the importance of active contribution and gaining in depth knowledge in advance.

Third, it is important that HCPs are prepared for the implications of the implementation of the toolkit. Both end users and HCPs believe that a nurse is the designated HCP to introduce, deliver and work with the toolkit because this professional has both the time and expertise. Moreover, both end users and HCPs express that successful implementation of the toolkit depends on the attitude and behaviour of doctors in general and medical specialists in particular. They need to support the use of the toolkit and need to centralize patients and ICs in both their conversations and decision making.

Finally, it is important to create the right conditions in healthcare to implement the toolkit. End users also state that it is important that the toolkit is affordable for everyone and recommend determining a price and organising a rebate for example via health insurance companies or patient organisations. HCPs express that it is important to first integrate the toolkit in their own work process and in the registration systems of their organisations (See Appendix B).

## 4 Discussion and conclusion

### 4.1 Discussion

This qualitative study explored the perceived acceptability of the prototype toolkit MijnBlik according to end users and HCPs. It sheds light on the perceived appreciation and practical value of this prototype toolkit and give directions for further implementation of the toolkit in healthcare. End users and HCPs highly appreciate the inviting appearance of the hardcopy version of the prototype toolkit and the four topics that get to the heart of the matter, although there is less appreciation for the website and the fact that the content of the prototype toolkit is too extensive. End users and HCPS are ambivalent about the usability of the prototype toolkit especially in relation to certain subgroups of end users. They are very positive about the practical value of the prototype toolkit and believe that both end users and HCPs may benefit from the prototype toolkit because it may strengthen the power and control of end users and support HCPs in tailoring communication and care to end users. End users and HCPs recommend further development of the hardcopy version of the prototype, provide more support for HCPs in using the toolkit and arranging several facilitating conditions at different levels of the care process before the toolkit can be evaluated and implemented in clinical practice.

#### 4.1.1 The toolkit is useful but needs some adjustments to improve it's perceived ease-of-use

Based upon the expressed practical value of both end users and HCPs the prototype toolkit seems to be perceived as useful. Participants perceive that the toolkit can be used by both end users and HCPs. End users perceived that the toolkit could be used during the whole disease trajectory of COPD and CHF and also by other end users not just those with COPD and CHF. It is positive that participants perceive the toolkit as useful because according to the TAM there is a direct relationship between PU and actual use [28]. However, the toolkit needs to be adjusted prior to further evaluation and implementation because the content of the toolkit is experienced as being too extensive, the hardcopy version is experienced as being incomplete without the website and the website is experienced as being too complicated to use. Due to the direct relationship between PEOU and PU, based upon the TAM, it is important to reduce the number of tools, to make it possible to use the hardcopy version without the website, and to include easy to use online tools on the website. Besides adjusting the prototype toolkit based upon the findings of this study, it is also important to deliver the toolkit early in the disease trajectories when end users may still not have discussed everything with their HCPs.

#### 4.1.2 Hardcopy version is more appreciated than the website

This study revealed that the hardcopy version of the toolkit is more appreciated than the website. Both end users and HCPs expressed that the website may be a problem for older adults and more severely ill patients. However, age and disease severity are not the most important factors in relation to the ability to use the internet. More important are the socio-economic status and the level of (e)Health literacy [[34], [35], [36]]. This may mean that it remains important to use different modes of delivery of the toolkit in order to address the needs of different types of individuals within the population of end users [34,35]. It also may mean that the prototype toolkit needs further development in order to increase the usability for end users with low levels of (e)Health literacy which is highly prevalent among patients with COPD and CHF [[37], [38], [39]]. Another implication of this finding is that individual differences need to be included as one of the determinants of PU and PEOU in future studies about the acceptability of the prototype toolkit.

#### 4.1.3 HCPs uncertain about timing and content phasing of the prototype toolkit

HCPs expressed their concerns that the content of the prototype toolkit does not match the disease course of COPD and CHF. This was particularly the case in relation to the theme “talking about the future”. HCPs were uncertain about the timing and content phasing of the prototype toolkit because they were concerned about emotional burden on end users and the impact on their relationship with end users [40,41]. However, participating patients recommended delivering the complete toolkit as early as possible in the disease process although one patient, recently diagnosed with CHF, had been confused for several days after receiving the toolkit. This difference in perspective between HCPs and end users can be an illustration of social influences like subjective norms as determinants of PU and PEOU. Although it is recommended to integrate PC and Advanced Care Planning (ACP) early in the disease trajectory of patients with COPD or CHF [1,2,42,43] the readiness differs from person to person and is based upon individual preferences and disease severity [[44], [45], [46], [47]]. This implies that timing and content phasing of the toolkit needs to be tailored to each individual end user and HCP's need to be able to assess end users readiness and adjust the introduction of the toolkit and their communication accordingly [48,49]. Specific clinical indicators to initiate ACP conversations for patients with organ failure are: 1) after a period of illness, 2) after an exacerbation, 3) in a period with relative wellness, 4) in an appropriate setting with adequate time and presence of a family member [50]. These indicators can be helpful to HCPs in introducing and delivering the prototype toolkit in a timely manner to end users.

#### 4.1.4 Context matters in relation to the implementation of the prototype toolkit

The high practical value given by both end users and HCPs indicates that the toolkit meets their needs. However, the intended use of the toolkit in clinical practice does not only depend on the system characteristics of the prototype toolkit It became clear that this also depends on the integration of the toolkit in the work process of HCPs, the registration systems of healthcare organisations and the financial rebate of the toolkit. These facilitating conditions need to be established prior to the evaluation and implementation of the prototype toolkit in clinical practice [18,20,23,51]. Further research of facilitating conditions is needed before the prototype toolkit can be implemented in clinical practice.

#### 4.1.5 Strengths and limitations

One strength of this study was the inclusion of both end users and HCPs. This provided insight into the perspectives of intended receivers and intended delivers of the toolkit. It also made it possible to analyse similarities and differences in their perspectives. The inclusion of end users with varying time spans since diagnosis was another strength because the perceived ease of use and practical value of the toolkit may depend on the disease duration.

A limitation of this study was the existence of selection bias among both end users and HCPs. More motivated and empowered end users were included in this study probably due to the recruitment approaches used due to Covid-19. Although the intention was to recruit an equal number of patients and ICs, more patients than ICs were included. The consequence of this is that the perspective of ICs is insufficiently addressed. The majority of the HCPs were female nurses, there were only two GPs and no medical specialists. This implies that the perspective of male HCPs, GP and medical specialists, needs to be further assessed. Finally, the perceived and not the experienced acceptability of the prototype toolkit during conversations in clinical practice was evaluated in this study due to Covid-19.

### 4.2 Innovation

The research presented in this article outlines a qualitative evaluation of the acceptability of a prototype toolkit for patients with COPD or CHF and their ICs. The aim of the prototype toolkit is to empower patients and ICs to express their perspective in palliative care conversations with their HCPs [15]. The use of a Design Thinking (DT) approach, in which we examined the perceived acceptability of the toolbox in this study prior to the evaluation and implementation of the toolkit, is an innovative approach in palliative care research [29,52]. This approach is based upon the active engagement of relevant stakeholders in the development, evaluation and implementation of healthcare interventions [29,52]. The use of DT can result in a more useful, acceptable and effective toolkit compared to the use of less iterative and user-centred methods [52]. The evaluation of the acceptability of the toolkit by patients, ICs and HCPs can contribute to a succesfull implementation of the toolkit and its effectiveness and it does justice to the practical reality in which palliative care conversations are performed [29].

### 4.3 Conclusion

The high appreciation of especially the hardcopy version and the practical value are positive indicators of the perceived acceptability of the prototype toolkit. However, further development and testing of the prototype toolkit is required to increase itsacceptability by end users and HCPs. Since acceptability does not only depend on the system characteristics of the toolkit itself, it is also important to support HCPs before and during implementation, to establish facilitating conditions in healthcare and further study system characteristics, facilitating conditions, individual differences and social influences as determinants of PU and PEOU prior to the evaluation and implementation of the toolkit in clinical practice.

## CRediT authorship contribution statement

**Annet Olde Wolsink-van Harlingen:** Writing – original draft, Visualization, Methodology, Investigation, Formal analysis, Data curation. **Jan Jukema:** Writing – review & editing, Supervision. **Kris Vissers:** Writing – review & editing, Supervision. **Madeleen Uitdehaag:** Writing – review & editing, Supervision, Funding acquisition, Formal analysis, Conceptualization. **Jeroen Hasselaar:** Writing – review & editing, Supervision. **Leontine Groen-van de Ven:** Writing – review & editing, Project administration, Methodology, Investigation, Formal analysis, Data curation.

## Declaration of competing interest

None.

## Figures and Tables

**Table:** 

Research question	Topics	Main questions and sub-questions
	***Introduction to topics concerning experience with the toolbox***	*You have received MijnBlik from us and have been able to look at it.* *We are very curious about your experiences with MijnBlik.* *The first questions are about that.*
1 and 2	First impression of the prototype toolkit MijnBlik	**What was your first impression of MijnBlik (tin box and website)?**
		- Did this match your expectations? - Why yes/no?
1	Experience with using the prototype toolkit MijnBlik	**What have you done with MijnBlik (tin box and website) since receiving it?**
		- How have you used the tin box/website? - What is it like to use the tin box/website? - How do you find using the tin box? - How do you find using the website?
2	Experienced effects	**What thoughts and/or feelings has MijnBlik evoked in you?**
		- How did you experience that?
	***Introduction to topics concerning opinion of prototype toolkit MijnBlik***	*We are also curious to know your opinion of MijnBlik: the tin box and the website.* *That's what the following questions are about.*
1 and 2	Opinion of prototype toolkit MijnBlik	**What do you think of MijnBlik (tin box and website)?**
		- What do you like about it? - What do you not like about it? - What are you missing in MijnBlik? - What do you find unnecessary about MijnBlik?
1 and 2	Experience with fans and conversation tools	**What do you think of the fans?**
		- Which fans did you look at in the tin box? - What made you look at them? - What made you not look at the other fans? - What do you think of the information on the cards with conversation tools?
		**What do you think of the conversation tools?**
		- Which conversation tools have you looked at? - What made you look at them/not look at them?
1	Opinion on obtained information	**What do you think of the information you have received?**
		- What do you think of the information on the explanation card included in the tin box? - What do you think of the information on the website?
	***Introduction to topics concerning the application (implementation) of the prototype toolkit MijnBlik***	*MijnBlik was created to help people with COPD and/or heart failure and their informal caregivers to have good conversations with their healthcare professional.* *That's what the next questions are about.*
2	Expected support of prototype toolkit MijnBlik	**What would you like to change or improve about the conversations with your healthcare professional?**
		- What challenges do you face in conversations with your healthcare professional? - What do you think you should mention to ensure that you receive good care? To what extent is this happening already?
		**How do you think MijnBlik could help you with this?**
		- Which parts of MijnBlik could help with this?
2 and 3	Applying the prototype toolkit MijnBlik in practice	**How do you think you will use MijnBlik to prepare for or conduct your next conversation with your healthcare professional?**
		- What do you expect to change in the conversations with your healthcare professional if you start using MijnBlik? - How do you expect your healthcare professional will react if you use (what you have learned from) MijnBlik during the consultation? - What requirements should the tin box/website meet in order to start/continue using it?
	***Introduction to the topic related to the costs of prototype toolkit MijnBlik***	*Finally, I would like to ask you some questions about the costs of working with MijnBlik in terms of time and money*
3	Cost of use (time and money)	- How much time have you spent on MijnBlik? - How much time would you like to spend on it? - How much would you be willing to pay for MijnBlik? - Who do you think should pay for MijnBlik?

**Table:** 

Research question	Topics	Main questions and sub-questions for the topics
	***Introduction to topics concerning opinions of the prototype toolbox MijnBlik***	*We are curious to know your opinion of MijnBlik: the tin box and website.* *That's what the next questions are about.*
1	Opinion of MijnBlik	- What do you like about MijnBlik? - What do you like less/not so much about MijnBlik? - What are your tips and tops for the tin box, the website, the toolkit, the tools?
	***Introduction to topics about the application (implementation) of MijnBlik***	*MijnBlik was created to help people with COPD and/or heart failure and their loved ones have a good conversation with their healthcare professionals.* *That's what the next questions are about.*
2	Expected support of the prototype toolkit MijnBlik	- What can you, as a healthcare professional, gain from using MijnBlik in the guidance of your clients and their loved ones?
3	Applying the prototype toolkit MijnBlik in practice	- What do you need to start using MijnBlik in guiding your clients and their loved ones? - What do you need to continue using MijnBlik in the long term? - What do you think are the inhibiting and promoting factors for using MijnBlik within your organisation?

**Table:** 

Research question	Theme	Category	Code	End users	HCPs
***Research question 1a*** What do end users and healthcare professionals **like** about the toolbox?	Form	Design is original and handy	Size is handy/easy to put down or away	+	+
			Shape is original	+	
			Material is durable	+	
			Website is easy to keep up to date	+	
		Looks inviting	Looks appealing and inviting	+	+
			Appearance is attractive and neat	+	+
			Attractive sets of cards	+	+
			Colours are attractive and fresh	+	+
		Lay-out is accessible	Clearly legible	+	+
			Titles and text are clear and concise	+	
			Contents are conveniently arranged		+
	Contents	Four topics get to the heart of the matter	Are relevant and topical	+	+
			Form the core	+	+
			Are appealing	+	
			Four themes are clear		+
			Affects all facets of healthcare		+
			Future-oriented focus		+
		Tools are appealing	Make use of what already exists	+	+
			Attractive presentation format	+	+
			Sound advice		
			Useful tools		+
	Use	Easy to use	Website is self-explanatory	+	+
			Tools are easy to find	+	+
			Instruction sheet is good introduction	+	
			Instruction sheet helps in selecting card wheel	+	
			Clear how tin box works	+	
		Can be used in a self-paced manner	You choose when you want to do something with it	+	+
			You choose whether you want to do something with it	+	
			You choose what appeals to you	+	
			Tin box is sufficient on its own	+	
			I only use the website	+	
		Broad application	Suitable for end users and healthcare professionals	+	+
			Suitable for all stages of disease	+	
***Research question 1b*** What do end users and healthcare professionals **dislike** about the toolbox?	Form	Tin box is too big	Doesn't fit through letterbox	+	
			Doesn't fit easily in bag (hand/work bag)		+
		Tin box incomplete without website	Only contains references to websites	+	
			You can't do anything with the tin box on its own	+	
			Use is limited without website		+
		Website reduces usability	The toolbox is dependent on internet	+	+
			Not all older adults use a computer	+	+
			Large portion of target group still not internet users	+	+
			Searching on the internet too much trouble for vulnerable patients	+	+
			Keeping website up to date takes up a lot of time		+
	Contents	It is too comprehensive	A lot of information	+	+
			A lot of information repetition	+	
			Many tools		+
			Rather extensive		+
			Easy to get bogged down		+
			Can be experienced as overwhelming/risk of overload		+
		Does not meet needs	No need for in-depth information (need for practical information)	+	
			No desire to look at website (want to continue thinking for themselves)	+	
			Everything has already been discussed with healthcare professionals	+	
			Certain tools are for healthcare professionals	+	
			We already pay a lot of attention to self-management		+
		Sequence of themes does not correspond with course of diseases	Talking about the future is too heavy at first		+
			Themes 2 and 3 correspond to the start of the disease		+
			Themes 1 and 4 are suitable for the final stage		+
			Terminal stage possibly under-emphasised		+
	Use	Too difficult and complex	Difficult to browse through card wheel	+	+
			Confusing that websites are from other providers	+	+
			Easy to get lost in underlying websites	+	
			Difficult to return to homepage	+	
			It is quite complicated		+
		Takes too much time and effort	It takes too long	+	+
			Continually have to search on the computer	+	+
			Having to click too much on website	+	+
			Getting computer requires extra action		+
			Introducing the toolbox takes a long time		+
			Printing and discussing tools is too labour intensive		+
		Too much is still unclear	Unclear who it is intended for		+
			Is it suitable for everyone?		+
			Are we reaching the right target group?		+
			Does the group we want to reach benefit from this?		+
			Unclear what you are specifically going to do with it		+
			Unclear who should refer to it in discussions		+
***Research question 2*** What practical significance do end users and HCPs experience or expect from using the toolbox?	Strengthens power and control of end users	Provides insight	Into the disease and disease process	+	+
			Into healthcare options	+	+
			How to have a conversation with your healthcare professional	+	
			Into how you can organise your own future	+	
			Provides an overview when discussing the future	+	
		Creates awareness	What you can think about	+	+
			What you can ask and who you can ask	+	+
			What you can discuss	+	+
			What is important to discuss	+	+
			How to interact with your healthcare professional	+	
			How to converse with your healthcare professional	+	
			What you can do yourself		+
			What are your wishes and concerns		+
			What preoccupies you		+
		Being confronted brings feelings and thoughts to the surface	Thinking about your own future is confronting	+	+
			Felt confused for a few days	+	
			This is not about me	+	
			Fear can increase		+
			Can be experienced as interfering		+
			End user can shut themselves off even more		+
			Can create unease		+
			Can take away hope		+
		Makes you think	About the conversation with the healthcare professional	+	+
			About the future	+	+
			About end of life	+	
		Provides incentive to act	Have conversations with patient, relatives or fellow sufferers	+	+
			Prepare conversation healthcare professional	+	+
			Change daily structure	+	
			Ask partner for help	+	
			Request folders/booklets	+	
			Complete and issue a living will	+	
		Supports dialogue between patient and relatives	Better conversations with each other	+	+
			Understanding each other better	+	+
		Supports conversation with healthcare professional and decision-making	Asking the right questions		+
			Making things open to discussion		+
			Making the right decisions		+
	Supports healthcare professionals to align with end users	Supports dialogue	Jointly responsible for content of conversation	+	+
			Easier to talk to each other		+
			More openness and depth		+
			More active participation end users		+
		Contributes to strengthening control of end users	Let end users have more control		+
			More emphasis on self-management		+
		Helps to tailor healthcare	Able to act on healthcare needs		+
			Able to deliver appropriate healthcare		+
***Research question 3*** What do end users and healthcare professionals recommend for implementing the toolbox in the healthcare system?	Further development of toolbox	Adjusting contents	Make suitable for a broader target group	+	+
			Make simpler and more convenient	+	+
			Make tin box useable without website	+	+
			Do not include websites from other providers	+	+
		Alter the shape	Laminate box contents	+	
			Make box smaller and more compact		+
	Inform, instruct and support healthcare professionals in advance	Need for more information	Reason for developing toolbox		+
			Who will hand out the toolbox?		+
			Who will receive the toolbox?		+
			When can you hand out the toolbox?		+
			What are you going to hand over?		+
			What you do with it after handing it over		+
			How do you discuss this?		+
			What should we do and what should we not do?		+
		Need for preparation and support	First, become familiar with it yourself, as healthcare professional		+
			Support in getting started		+
		Prepare for introduction of toolbox	First build a trusting relationship	+	+
			Clarify importance of active contribution		+
			Clarify importance of gaining in-depth knowledge in advance		+
	Prepare healthcare professionals for implications	Nurse is designated person to work with toolbox	Has the relevant expertise	+	+
			Has the time	+	+
			Doctors do not want to give explanation	+	+
			Doctors do not have time for giving explanation	+	+
		Doctor supports use of toolbox	Has supporting attitude	+	+
			Takes the time needed	+	
			Goes along with it		+
		Doctor puts end user at centre of discussions and decision-making	Not only the doctor decides	+	
			Have discussions from perspective of what is important to end user		+
			Create space for end users' own choices		+
	Create conditions in healthcare	Determine asking price and organise rebate	Must be affordable to everyone	+	
			Maximum between 10 and 20 euros	+	
			Must be provided free of charge	+	
		Integrate toolbox in healthcare process	Embed at polyclinic		+
			Let it become part of the treatment plan		+
			Good cooperation with the first line (earlier re-referral)		+
		Integrate use into registration systems	We are now being judged on production figures		+
			Do not write off use of MijnBlik in the district		+
			Build into digital systems		+
